# Network-based identification and prioritization of key transcriptional factors of diabetic kidney disease

**DOI:** 10.1016/j.csbj.2022.12.054

**Published:** 2023-01-02

**Authors:** Ikhlak Ahmed, Mubarak Ziab, Sahar Da’as, Waseem Hasan, Sujitha P. Jeya, Elbay Aliyev, Sabah Nisar, Ajaz A. Bhat, Khalid Adnan Fakhro, Ammira S. Alshabeeb Akil

**Affiliations:** a Department of Human Genetics-Precision Medicine in Diabetes Prevention, Precision Medicine Program, Sidra Medicine, P.O. Box 26999, Doha, Qatar; b Zebrafish Functional Genomics, Integrated Genomic Services Core Facility, Research Branch, Sidra Medicine, P.O. Box 26999, Doha, Qatar; c College of Health and Life Sciences, Hamad Bin Khalifa University, P.O. Box 34110, Doha, Qatar; d Department of Genetic Medicine, Weill Cornell Medical College, P.O. Box 24144, Doha, Qatar; e Human Genetics Department, Laboratory of Genomic Medicine-Precision Medicine Program, Sidra Medicine, P.O. Box 26999, Doha, Qatar; f Department of Physiology and Biophysics, Weill Cornell Medical College, P.O. Box 24144, Doha, Qatar

**Keywords:** Diabetic nephropathy, Diabetic kidney disease, Gene expression, Network analysis, Hyperglycemic zebrafish, Transcription factors, DACH1, LMX1B, WT, Therapeutic biomarkers

## Abstract

Diabetic nephropathy (DN) is one of the most established microvascular complications of diabetes and a key cause of end-stage renal disease. It is well established that gene susceptibility to DN plays a critical role in disease pathophysiology. Therefore, many genetic studies have been performed to categorize candidate genes in prominent diabetic cohorts, aiming to investigate DN pathogenesis and etiology. In this study, we performed a meta-analysis on the expression profiles of GSE1009, GSE30122, GSE96804, GSE99340, GSE104948, GSE104954, and GSE111154 to identify critical transcriptional factors associated with DN progression. The analysis was conducted for all individual datasets for each kidney tissue (glomerulus, tubules, and kidney cortex). We identified distinct clusters of susceptibility genes that were dysregulated in a renal compartment-specific pattern. Further, we recognized a small but a closely connected set of these susceptibility genes enriched for podocyte differentiation, several of which were characterized as genes encoding critical transcriptional factors (TFs) involved in DN development and podocyte function. To validate the role of identified TFs in DN progression, we functionally validated the three main TFs (DACH1, LMX1B, and WT1) identified through differential gene expression and network analysis using the hyperglycemic zebrafish model. We report that hyperglycemia-induced altered gene expression of the key TF genes leads to morphological abnormalities in zebrafish glomeruli, pronephric tubules, proximal and distal ducts. This study demonstrated that altered expression of these TF genes could be associated with hyperglycemia-induced nephropathy and, thus, aids in understanding the molecular drivers, essential genes, and pathways that trigger DN initiation and development.

## Graphical Abstract

**Figure:**
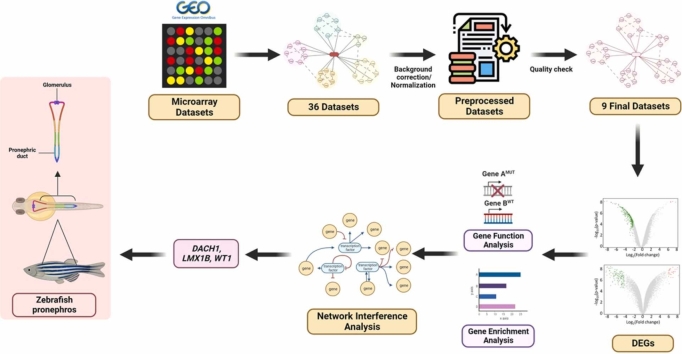

## 1 Introduction

Diabetic nephropathy (DN), also known as diabetic kidney disease, is a main microvascular complication of diabetes mellitus (DM) and a key factor in increased morbidity and mortality in DM patients. Several factors, including hyperglycemia-generated metabolic fluctuations [1], age at onset [2], DM duration [3], hypertension [4] and genetic susceptibility [5], have been proven as significant contributors to the disease progression. It is majorly the glomerular damage, besides tubulointerstitial fibrosis, contributing to the pathogenesis of DN [6], [7]. The current treatment for DN is controlling glucose levels and reducing systemic blood pressure using renin-angiotensin system inhibitors [8]. However, even with these therapeutic options, the prevalence of DN kept arising, and innovative treatment strategies were immediately required.

Recently, multiple studies have used computational approaches to identify risk factors that could contribute to DN pathogenesis [9], [10], [11], [12]. An enormous volume of data, including high throughput omics data and clinical information collected through Electronic Health Records, has been produced because of the tremendous advancements in biotechnology and the health sciences. Application of appropriate computational methods including machine learning and meta-analysis approaches is essential to transform all this information intelligently into useful knowledge. Finding new molecular signatures requires integrating multiple datasets with greater sample sizes that are less prone to systematic biases. These signatures may offer additional insights and treatment options into the etiology and pathogenicity of DN.

The hyperglycemic conditions in DM patients stimulate several molecular pathways, and biochemical reactions in diverse kidney cell types. These include activation of protein kinase C [13], increasing inflammatory mediators and growth factors [14], the elevation of complex glycation end products resulted from nonenzymatic reactions [15], and the accumulation of reactive oxygen species (ROS) [16]. Nonetheless, a comprehensive understanding of the gene regulation mechanisms that aggravate hyperglycemia-arbitrated biochemical imbalances is less understood. In vitro and in vivo research investigations in mesangial and glomerular cells, podocyte loss and epithelial dysfunction have emphasized several transcription factors (TFs) that contribute to regulating gene expression in diabetic contexts [17], [18].

For instance, the transcription factor NF-κB is activated by ROS cellular signaling or through hyperglycemia-induced cytokines influencing the glomeruli microinflammation [19]. The SMAD proteins, including SMAD1 and SMAD3, are activated in response to transforming growth factor (TGF- β) and glycation end products to maximize the transcriptional activity of mesangial cells through the production of extracellular matrix proteins [20], [21], [22]. Increasing angiotensin II in glomerular mesangial cells and elevated glucose levels activate STAT1 and STAT3, leading to the production of extracellular matrix proteins and manipulating cellular growth [23], [24].

Transcriptional dysregulation in the DN can occur at several levels, including modifications in upstream cellular signals or the TF itself. In this regard, TFs are the final founder proteins at the site of gene regulation to arbitrate the cellular responses to pathogenic elements in DM. Consequently, TFs may represent attractive therapeutic targets that could avoid many exertions with signal idleness and unwanted signal transduction events often faced in therapies designed to target the upstream signaling pathways [24], [25].

Despite the advancement in this topic, obstacles in developing TFs as drug molecules are still challenging. For instance, several TFs are directly regulated by synthetic steroid ligands such as estrogen receptor in endocrine treatment for several cancers [26] and glucocorticoid receptor in immunosuppression therapy [27], [28]. However, for some other TFs, direct targeting designs exist, e.g., c-Myc expression targeting and regulation through quadruplex stabilizer [29], [30], [31], disrupting STAT3– or NF-κB–DNA interactions by decoy oligonucleotides [32], [33], and small interfering RNA or antisense oligonucleotide technology for many other diseases [34], [35]. Given that, more realistic strategies for targeting TFs are essential.

An example of a therapeutic approach for DN is through induction of *O*-GlcNAcylation of carbohydrate response element (ChRE)-binding protein (ChREBP) to high glucose levels in mesangial cells [36]. It is a basic helix–loop–helix/leucine zipper TF produced in several metabolically related tissues, such as pancreatic β-cells, liver, and adipocytes, and acts as a moderator of glucose-responsive gene activation [36], [37], [38]. Following glucose stimulation, ChREBP translocates into the nucleus, thus producing a heterodimer with Max-like protein X (Mlx) that conjugate to the carbohydrate response element (ChRE) for transcriptional regulation of genes related to glucose, fatty acid steroid and lipid metabolism [38], [39], [40], [41], [42].

Despite the druggability hurdle yet to be solved, progress is being achieved in research targeting the modulation of TFs with possible clinical advantages. To create novel therapies for DN, new research studies and concepts are needed to determine specific TFs and investigate their potential functional role in the disease pathogenesis. This study aims to identify key kidney-specific TFs of DN using publicly available microarray datasets and further prioritize them through functional validation in hyperglycemic zebrafish models. The study also discussed the specific role of each identified TF in developing DN to their correlated functions.

## 2 Methods

### 2.1 Dataset selection and curation

All microarray datasets used in this study were retrieved and accessed using the Gene Expression Omnibus [43] repository on January 25, 2022. The search keywords were limited to “diabetic nephropathy”, and the results were further filtered based on organism (“Human”) and study type (“Expression profiling by array”). The resulting entries (n = 36) were further manually curated based on the following criteria: (1) relevance to DN; (2) availability of data for kidney tissue; (3) classification of study as observational; and (4) availability of baseline comparators, i.e., non-chronic or acute kidney disease tissue.

### 2.2 Individual dataset pre-processing and quality-check

The final curation of microarray datasets (Summarized in Table 1) was processed using a uniform “*R*” [44] pipeline script. Individually selected raw intensity expression (CEL) files obtained based on the criteria mentioned above were imported into R. The background expression level was subtracted, quantile-normalized, median-polished, and log_2_-transformed using the *oligo* package version 1.58.0 [45]. The presence of duplicate samples was screened for using the *Dopplegang R* package version 1.22.0 [46], based on “expression” doppelgängers. If doppelgängers were found, they were manually removed based on further inspection of all information submitted for the suspected samples and the respective GEO series entries.

**Table 1:** List of GEO datasets curated and used for this study. All datasets were screened and obtained from Gene Expression Omnibus (GEO).

GEO Series	Platform	Tissue studied	Compared groups	Curated samples	Outliers (n)	Analyzed samples (n)	Sample-type breakdown (n of DN/n of Control)
GSE1009	GPL8300 (HG_U95Av2)	Glomerulus	DN; control	6	0	6	6^✧^
GSE30122^#^	GPL571 (HG-U133A_2)	Glomerulus; Tubulointerstitium	DN; control	Glom = 22 Tubu = 22	Glom = 1 Tubu = 1	Glom = 21 Tubu = 21	Glom = 8/13 Tubu = 10/11
GSE96804	GPL17586 (HTA-2_0)	Glomerulus	EDN; LDN; control	61	0	61	41/20^⁑^
GSE99340^#^	GPL19109 (HG-U133_Plus_2)	Glomerulus	DN	7	0	0^⁑^	0
	GPL19184 (HG-U133A)	Glomerulus; Tubulointerstitium	DN; control	29	1	14^❋^	15
GSE104948^#^	GPL22945 (HG-U133_Plus_2)	Glomerulus	DN; control	Glom = 7 Tubu = 22	0	Glom = 0 Tubu = 22	7/18
	GPL24120 (HG-U133A)	Glomerulus	DN; control	8	1	7	4/3
GSE104954	GPL22945 (HG-U133_Plus_2)	Tubulointerstitium	DN; control	25	2	23	6/17
	GPL24120 (HG-U133A)	Tubulointerstitium	DN; control	13	0	13	10/3
GSE111154	GPL17586 (HTA-2_0)	Kidney cortex	EDN; control	8	1	7	4/3

Abbreviations: EDN, early diabetic nephropathy; Glom, Glomerulus; LDN, late diabetic nephropathy; Tubu, Tubulointerstitium.

✧ Two samples had technical replicates.

⁑ EDN and LDN were both categorized as DN due to a lack of annotation details associated with the public dataset, despite an attempt to personally communicate with authors to attain further details.

✻ Glomerulus samples did not contain controls and were thus not analyzed.

# Not included in Network inference analysis

To determine sample quality scores for use in outlier detection, we computed array quality metrics [47] and the IQRray statistic [48]. To inform decisions to remove outliers, IQRrays were tested for outlier values using either Rosner’s or Dixon’s test, as implemented in *EnvStats* [49]. In addition, each sample was further inspected based on the quality scores from the *ArrayQualityMetrics* output and inspected visually based on principal component analysis (PCA) plots and density plots and based on outliers for distances between arrays using the Kolmogorov–Smirnov statistic *Ka* (boxplot outliers).

To functionally annotate probes, the *AnnotationDbi* package [50] was used to assign gene symbol keys to the probes according to the database that matched the platform used for each study. Datasets were further filtered to remove internal control probes and probes that matched to no or multiple gene symbols. Finally, expression values for multiple probes mapping to the same gene were averaged to obtain the mean.

### 2.3 Differential gene expression analysis and filtering for “core” differentially expressed genes

To test for differentially expressed genes (DEGs), we constructed least-squares fitted linear models for the expression data of each gene, and then we computed the log-fold changes in gene expression based on empirical Bayes function using *limma* [51]. This analysis was computed for all individual datasets separated by platform and kidney tissue (glomerulus, tubules, and kidney cortex) and for each of three “combined meta-analysis” datasets, including glomerulus and tubules datasets. The combined meta-analysis dataset samples that passed quality checks were combined into a single data frame, and the expression data were limited to genes that overlapped all individual datasets, which were corrected for batch effects using the *ComBat* [52] function in the SVA package [53]. DEGs were corrected for multiple testing at a false discovery rate (FDR) threshold of 5% using the Benjamini–Hochberg method [54], and the final list of genes was visualized for among-datasets highest-degree.

To screen for “core” DEGs between DN and control samples, we employed a vote-counting [55] approach using individual datasets for glomeruli and tubules and the combined meta-analysis dataset. First, statistically significant genes that passed a log_2_ fold-change threshold of 1.5 (raw FC ≈ 2.83) were retained; otherwise, they were dropped. This more stringent fold-change cutoff allowed us to isolate a panel of genes that varied widely in their gene expression among datasets and represented biologically meaningful DEGs [56]. These genes were further used to pick the topmost representative genes by vote-counting genes that were significant and passed the 1.5 log_2_ fold-change threshold. Genes that were vote-counted at least twice in either all the glomeruli or tubule datasets were used, and then the fold-change values from these genes were used to generate a heatmap using *pheatmap* [57]. The “Ward 2″ error sum of squares hierarchical clustering method [58] was used ad hoc to create clusters based on dissimilarity patterns across datasets. Finally, to determine the directionality of consistency (i.e., upregulation vs downregulation) for gene expression, the MetaVolcanoR package was used to calculate meta-analysis fold-changes, p-values, and sign consistencies for each of the genes using individual series datasets, calculated as implemented in MetaVolcanoR using the vote-counting and -combining approaches [59].

### 2.4 Gene function and disease enrichment analyses

The DEGs within the resulting heatmap clusters (which we term “aggregates” to prevent confusion with reference to the network analysis clusters generated below) were used to guide subsequent enrichment analyses. Subsequently, gene ontology (GO) and biological pathway (BP) sub ontologies and disease ontology semantic and enrichment (DOSE) terms that were enriched more than would be expected by chance were generated using *clusterProfiler* [60]. The enrichment functions were run using p- and q-value cutoff levels of 0.05, and p-values were adjusted *post-hoc* using the BH [54] method. To visualize the results, heatmaps, gene-concept networks (“*cnetplots*”), and treeplots were generated within *clusterProfiler*, and the number of viewable ontologies was adjusted to achieve meaningful visualization of results.

### 2.5 Network inference analysis

Network inference was used to explore and detect novel associations between the DEGs. The network inference algorithm identifies statistical dependencies between gene expression levels as edges. Specifically, we used network inference to infer the interactions between set of genes using the mutual information (MI) [61] measure. Statistically significant interactions were selected using a threshold of p < 10^−7^ and visualized using a force-directed layout in the software application Cytoscape [62]. This layout uses the MI values to determine the structure of the network. Genes with high MI values will appear closer in the network, whereas genes with low MI values will be further apart. Several highly correlated genes will appear as dense clusters in the network. A network clustering tool, MCODE [63], was used to find densely connected regions in the network. A protein-protein interaction database (STRING) was further used for functional enrichment of the clusters in the network and *in silico* validation of the gene interactions. This analysis was performed on a subset of our datasets (GSE30122, GSE99340, GSE104948 were not included in the Network inference analysis).

### 2.6 Functional validation experiments using Zebrafish model

#### 2.6.1 Animal care declaration

Zebrafish (*Danio rerio*) adults were maintained in a recirculating housing system under standard environmental conditions of temperature at 28 °C, conductivity at 1000 µs, and pH at 7.5 with 14 h light and 10 h dark cycle. All protocols used in these studies were approved by the local Animal Care and Use Committee and conform to the Zebrafish Policy published by the Qatar Ministry of Public Health that following the Guide for the Care and Use of Laboratory Animals published by the National Institutes of Health.

Zebrafish experiments were approved by the local Animal Care Committee (Qatar Foundation–EVMC-2020–006). Wild-type zebrafish were maintained as previously described in Da’as et al., 2020 [64]. Embryos were obtained from breeding and kept in an E3 solution at 28.5 °C. The zebrafish were divided into groups and raised in E3 media in control 0% glucose conditions or, to induce hyperglycemia in the developing zebrafish model, 2% high glucose conditions.

### 2.7 Zebrafish care, maintenance, and histological examination

Both groups were staged at 3 days old and fixed in 4% paraformaldehyde, then embedded in paraffin and cut into sections at a thickness of 4-µm. The kidney sections were stained with hematoxylin and eosin (H&E) as described previously [64]. Representative images were scanned using Phillips Ultra versatile scanner L60, at 40X magnification, scale bar, 50 µm.

### 2.8 Gene expression analysis of zebrafish

Total RNA was extracted from whole-zebrafish larvae using RNeasy mini kit (Cat. No. 74104, Qiagen, LLC, USA) according to the manufacturer’s instructions. Reverse transcription was achieved using Superscript IV (Cat. No. 18091050, Invitrogen, USA) as previously described [64]. Real-time quantitative PCR (qPCR) was performed in 20 μL reaction volumes using the listed primer sets **(**Table 2), and the qPCR reaction mixture contained 1 µL of cDNA, 4 µL of H2O, 1 µL of forward primer (10 µM), 1 µL of reverse primer, and 10 µL of PowerUp™ SYBR™ Green Master Mix (Cat. No. A25741). The qPCR reaction was carried out in a QuantStudio™ 12 K Flex Real-Time PCR System in a three stages standard curve experiment: (1) hold stage of 50ºC for 2 min, followed by 95ºC for 10 min; (2) fourty-cycle PCR stage of 95ºC for 15 s, followed by 60ºC for 1 min; (3) melt curve stage 95ºC for 95 s, 60ºC for 1 min, 95ºC for 15 s

**Table 2:** List of target genes and primers used to target zebrafish genes in the study.

**Target gene**	**5′ to 3′ Direction**	**Primer sequence**
*dachd*	Forward	TTCTCAAACACCTGGTCGGG
*dachd*	Reverse	CTGCTCCACGTTACACACCA
*lmx1ba*	Forward	CGCGCTGTTTTGTCTTGTCT
*lmx1ba*	Reverse	CACTGACTCTCTCCAGACGC
*lmx1bb*	Forward	GACTTGTGCACGGTGTAGGA
*lmx1bb*	Reverse	ACGTCCGAGACGTTTTCTCC
*wt1a*	Forward	TGCCTTTACCGTGCACTTCT
*wt1a*	Reverse	GCTGAACATCCTTGGTTGGC
*wt1b*	Forward	CGATGGGATCGGATGTCAGAG
*wt1b*	Reverse	GAGGCAGGGTGAAAGTCCA
*eefa1*	Forward	GGGCCCATGACATGACTACA
*eefa1*	Reverse	AGGTGACCAGTCGTAGTGGA

### 2.9 Statistical analysis

For expression analysis, the relative change in gene expression was quantified from the qPCR results using the Livak [65] method. The CT values for each gene in each experimental condition were assessed using 11–12 replicates in each group and were normalized to an internal control gene (*eefa1*). The relative expression was calibrated against the mean expression of control samples, and significant differential expression between groups was estimated using Wilcoxon rank-sum test. The data were analyzed for differential expression using custom scripts and the PCR [66] package in R.

## 3 Results

### 3.1 Identification of key differentially expressed genes

**Fig. 1:**
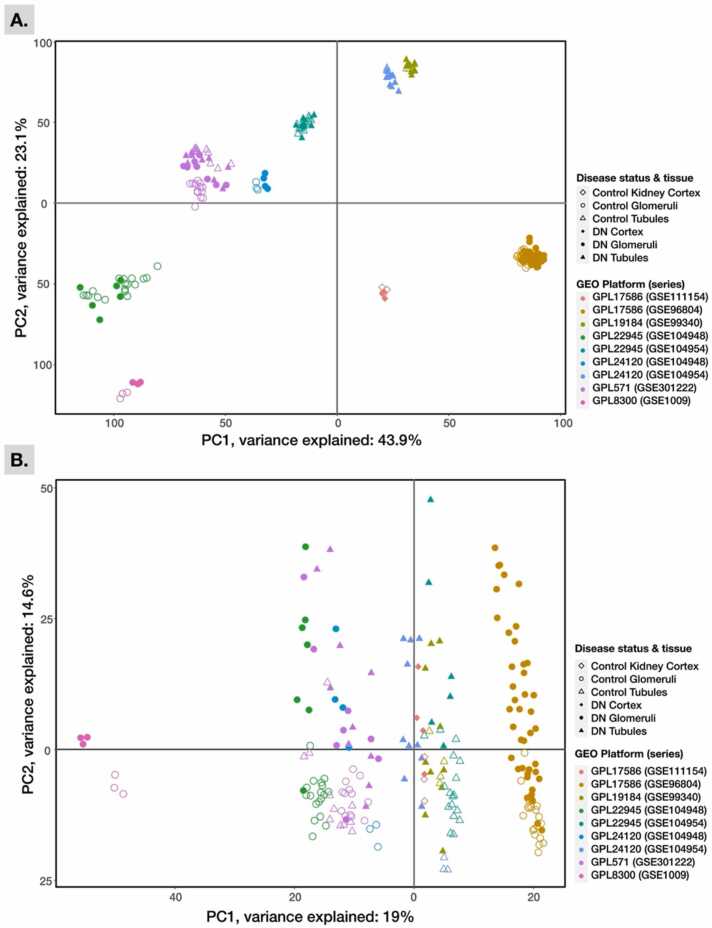
PCA of control and DN human kidney tissue datasets. Shown are the first two most variance-explaining principal components (PC) of RMA/log2-normalized and gene-level-averaged gene expression across all datasets (A) before or (B) after adjusting for batch-effects.

**Fig. 2:**
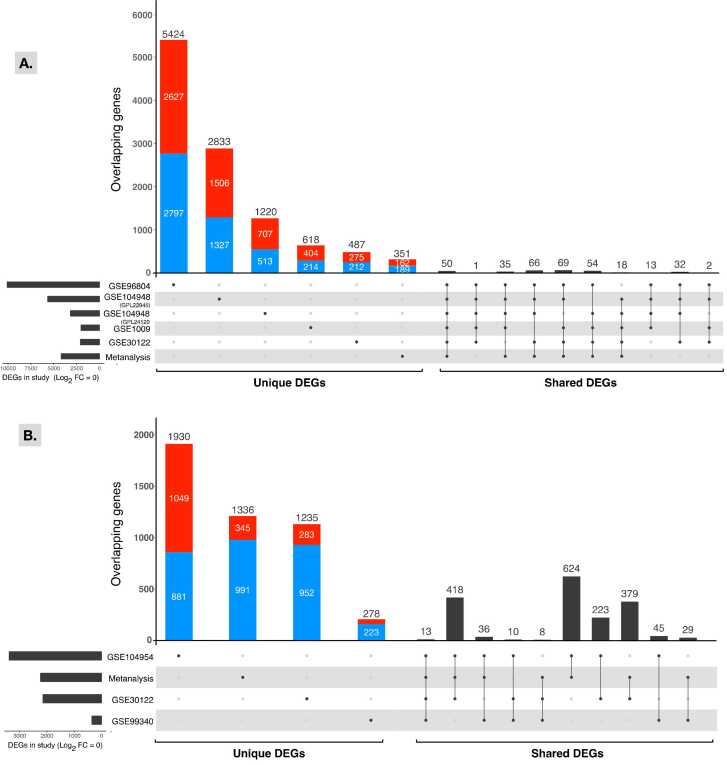
UpSetR plot of unique and shared DEGs among individual datasets. The number of total DEGs in each target glomerulus (A) and tubules (B) dataset is shown on the left side of the figure, and the left side of the figure shows the number of DEGs unique to each study, followed by the top 10 shared gene sets. Directionality of gene expression for unique genes is represented by red for upregulated genes and blue for downregulated genes.

In response to our GEO query, 62 DN glomeruli and 57 corresponding control samples, 36 tubulointerstitial and 35 corresponding control samples, and 4 DN kidney cortex and 3 corresponding control samples were selected for meta-analysis (Table 1). Considerable batch effects were observed across the preprocessed study samples, as illustrated in (Fig. 1A). The PCA plot of RMA/log_2_-normalized and gene-level averaged expression across all datasets indicated that platform-introduced experimental batch effects (represented by platform colors) largely contributed to the differences compared to sample origin and disease status. Using the empirical Bayes-based method *ComBat* [52], the undesirable batch effects on gene expression were minimized, as shown in (Fig. 1B). However, one dataset (GSE1009) remained separated from the other datasets. After batch effects were corrected, the amount of variance described by the first two principal components that explained the most variance also decreased, showing that the study samples were more homogeneous and that the bias resulting from uncontrolled cross-study experimental circumstances was less. Differential gene expression using least-squares fitted linear models (see Methods) was independently computed for each batch-corrected dataset. In general, the number of unique DEGs within each dataset was proportional to the total number of DEGs in the dataset (Fig. 2). For example, the dataset series GSE96804, which contained the highest number of DEGs (10,116), also contained the highest unique number of DEGs (5424) not found in other datasets. The same dataset was also one of the top five in the list of 10 datasets with DEGs overlapping with other datasets. Furthermore, fewer DEGs were found within the tubulointerstitial datasets than the glomerulus datasets. Finally, no DEGs were found between the DN and controls for the kidney cortical tissue samples (GSE111154) and one tubule dataset (GSE104954_GPL24120).

**Fig. 3:**
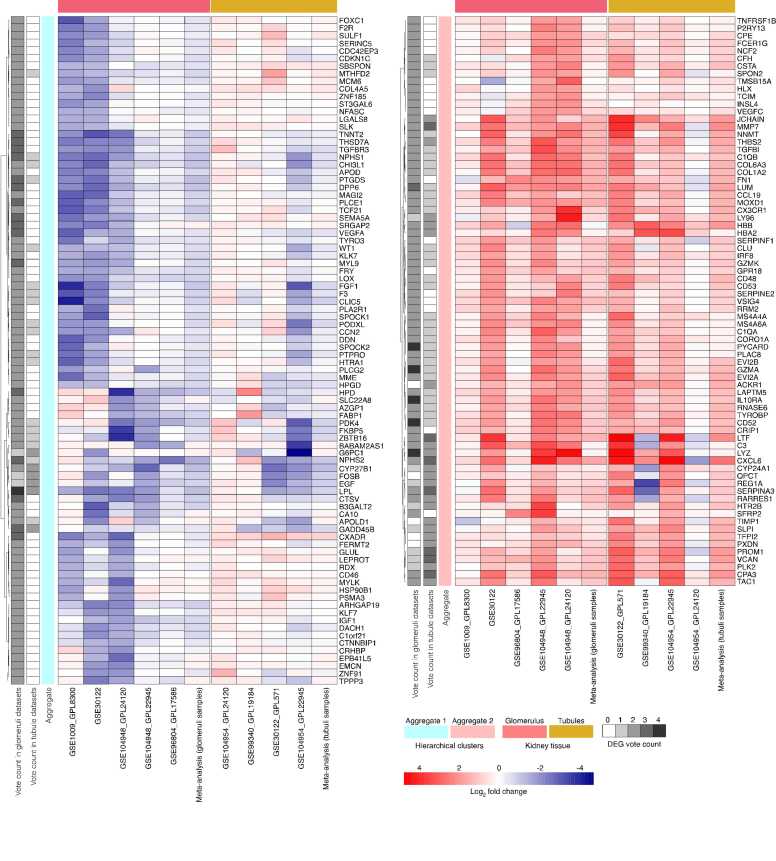
Heatmap of core DEGs human DN kidney tissue samples compared with healthy controls. To simplify visualization, the groups branching from the first hierarchical cluster dendrogram were split and situated side-by-side, labeled “Aggregate 1″ and “Aggregate 2″. The vote counts represent the number of times a gene was significantly down- or up-regulated (Log_2_ FC = 1.5, FDR<0.05).

We next used a vote-counting consensus approach to identify a subset of core DEGs. The dual approach using vote counts and meta-analysis fold changes allowed us to gain insight into concordances between the two methods and combine them to their fullest potential. Genes found in at least a third of the datasets and common to both glomerular and tubulointerstitial samples were retained for further pathway analysis. In total, 164 genes were identified as core DEGs between DN and control samples for either the glomerulus or tubule datasets. Out of the 164 core DEGs presented in the heatmap (Fig. 3), 147 were found to be DEGs in the glomerulus compartment (Table S1), compared to only 35 in the tubulointerstitium (Table S1). We broadly identified two aggregates of downregulated (Aggregate 1, n = 88 genes) and upregulated (Aggregate 2, n = 75 genes) sets of genes from the heatmap. These were used to conduct pathway enrichment analysis based on the consistency in the directionality of the gene expression changes [67].

### 3.2 Signature pathways in glomerular and tubular datasets

**Fig. 4:**
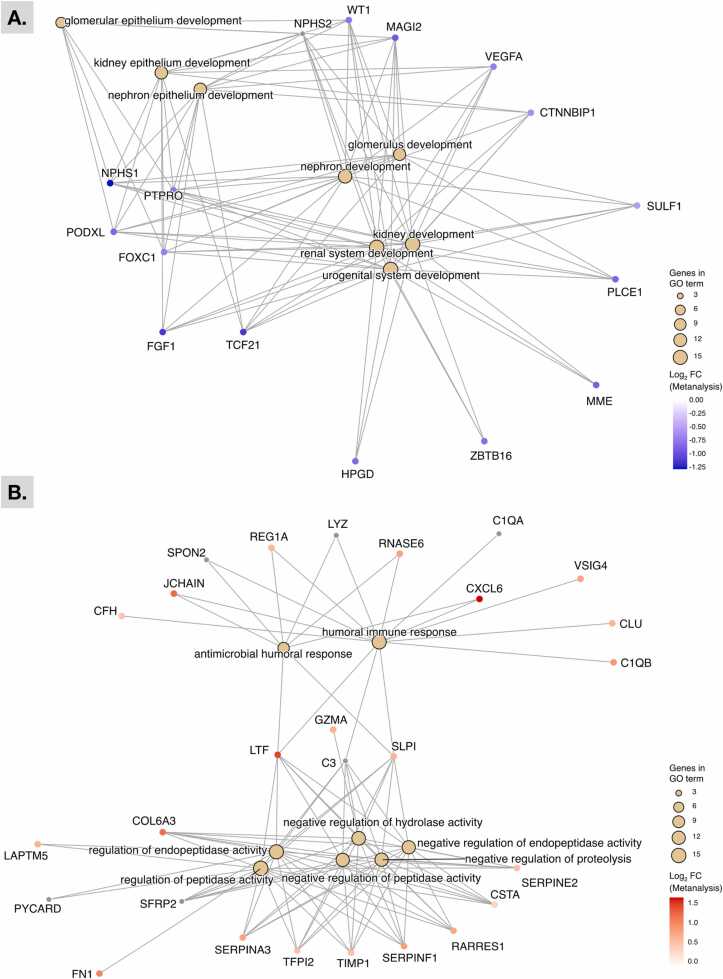
Gene-concept network plot comparing the functional profiles of DEGs in human DN kidney tissue samples compared with healthy controls. Shown are the top 8 gene-ontology terms assigned to genes from the first/downregulated (A) and second/upregulated (B) hierarchical cluster groups produced in Fig. 3. Log_2_ fold-changes were based on meta-analysis datasets using glomerulus or tubule datasets. Grey fold-change values indicate lack of data for meta-analysis dataset. Abbreviations: FC, fold change; GO, gene ontology.

**Fig. 5:**
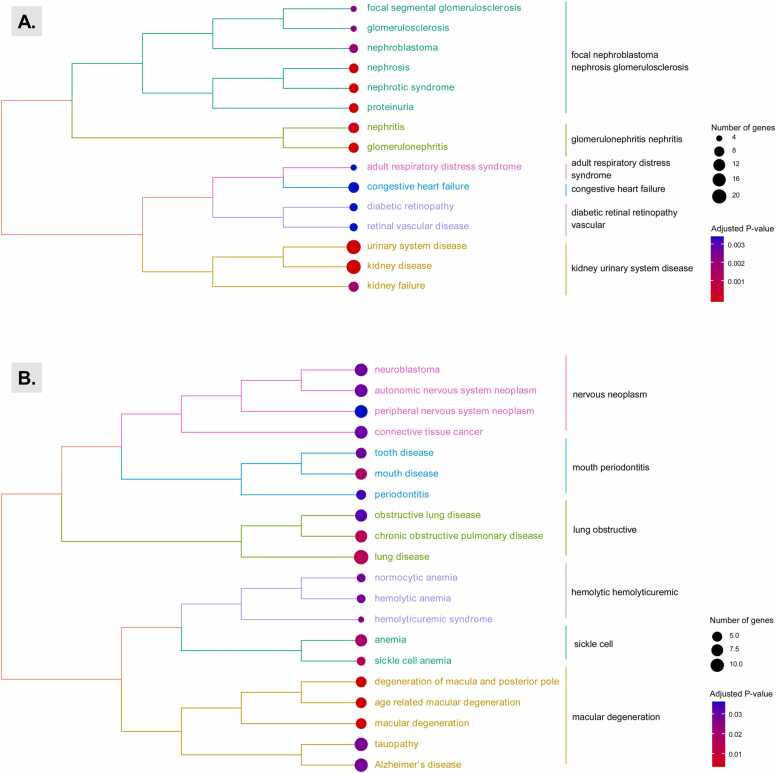
Tree plot of hierarchical clustering groups of disease ontology semantic and enrichment analysis terms for core DEGs. Each tree was created using the core DEGs defined and identified in the meta-analysis from Aggregate 1 (top) and Aggregate 2 (bottom).

The core downregulated DEGs in Aggregate 1 were found to be enriched for kidney-tissue development pathways (Fig. 4A). In contrast, the core upregulated DEGs in Aggregate 2 were heavily enriched for regulation (including negative regulation) of metabolic functions and humoral immune responses, with specific genes such as *LTF*, *SLPI*, and *C3* connecting both biological themes (Fig. 4B). In addition, kidney-related diseases and conditions were more highly enriched in the Aggregate 1 than the Aggregate 2 gene set. DEGs found within both clusters were also enriched for non-kidney related diseases, such as blood disorders (Fig. 5A) and respiratory illnesses (Fig. 5B).

### 3.3 Gene network inference

**Fig. 6:**
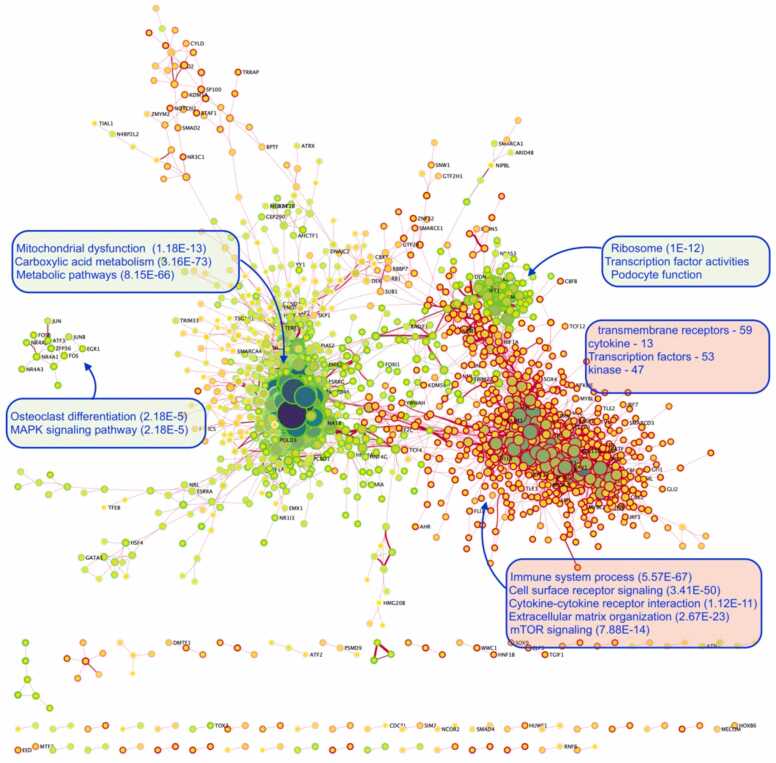
Overall network model using the most significant MI scores visualized by means of a force-directed layout, revealing several clusters in the network. The most prominent biological functions were used to annotate the clusters. Nodes in the network represent the genes, and edges (lines) represent the correlations, measured as mutual information values. Each node border color represents the fold-change in the gene expression in DN conditions. The node size represents the connection to other genes (hubs), and the thickness of the edges represents the strength of the association between genes.

We next looked at the interaction network model computed using the MI algorithm [61] and explored the top MI values between the gene pairs. The force-driven layout of the network showed intriguing associations between the clusters in the network. The STRING protein-protein interaction database (https://string-db.org/) was used in the annotation of these clusters based on the most prominent biological functions (Fig. 6). Four of the densest clusters in the network were enriched for podocyte function, immune system processes, mTOR signaling, cytokine receptor interactions, mitochondrial dysfunction, osteoclast differentiation, and MAPK signaling pathways. After assessing the sizes of the clusters, we focused on the podocyte differentiation cluster, which also consisted of high MI values (see Table 1).

### 3.4 Podocyte differentiation

**Fig. 7:**
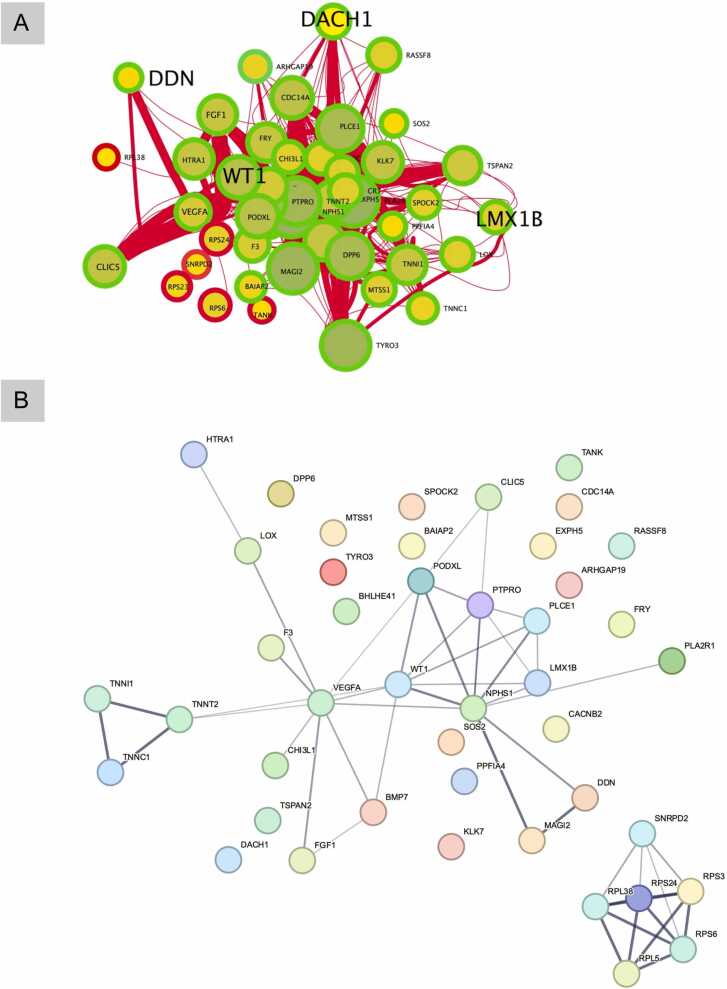
Network clustering of podocyte-differentiation-related differentially expressed genes in human DN kidney tissue compared with healthy controls. The size of each circle is proportional to the number of gene interactions, and the thickness of the edges represents mutual information between genes (A). Network cluster enriched for podocyte function with TFs in bold text. The interactions between the genes were also confirmed by STRING network (B).

Using network inference analysis, we found a sub-network cluster enriched for ribosomal activity and podocyte function, which was built from several hub genes and TFs known to play a role in podocyte differentiation (Fig. 7A). TF genes *DACH1* and *LMX1B*, which our DEG analysis showed to be significantly downregulated in DN, are reportedly critical to podocyte differentiation [68], [69]. These were connected to many other genes in the identified sub-network cluster and appeared as network hubs. The other hub genes in this cluster were NPHS1, TYRO3, VEGFA, PTPRO, PODXL, and FGF1, all of which were enriched in renal system development pathways (Fig. 3, aggregate 1). The expression fold changes for nearly all genes in this cluster was suppressed in DN compared to the controls. *NPHS1* showed interactions with 40 other genes in the network. STRING network analysis using the same gene sets supported several of these interactions and revealed *VEGFA*, *NPHS1*, and *WT1* as potential interaction partners. The protein-protein interaction network comprised of 44 nodes and 49 edges at an average node degree of 2.23 with a highly significant interaction enrichment (p-value = 5.41e-11), indicating that these proteins form at least a partially connected biological group.

### 3.5 Zebrafish functional validation

The TF genes *DACH1, WT1*, and *LMX1B*, which were downregulated in DN based on our DEG analysis, are master regulators of gene expression in podocyte differentiation. Additionally, they were identified as hubs with multiple potential interaction partners using our MI network inference model. To elucidate their gene regulatory functions, these three TF genes and their orthologs, *DACH1* (dachd), *LMX1B* (lmx1ba and lmx1bb), and *WT1* (wt1a and wt1b), were selected for additional functional validation in hyperglycemic zebrafish models.

**Fig. 8:**
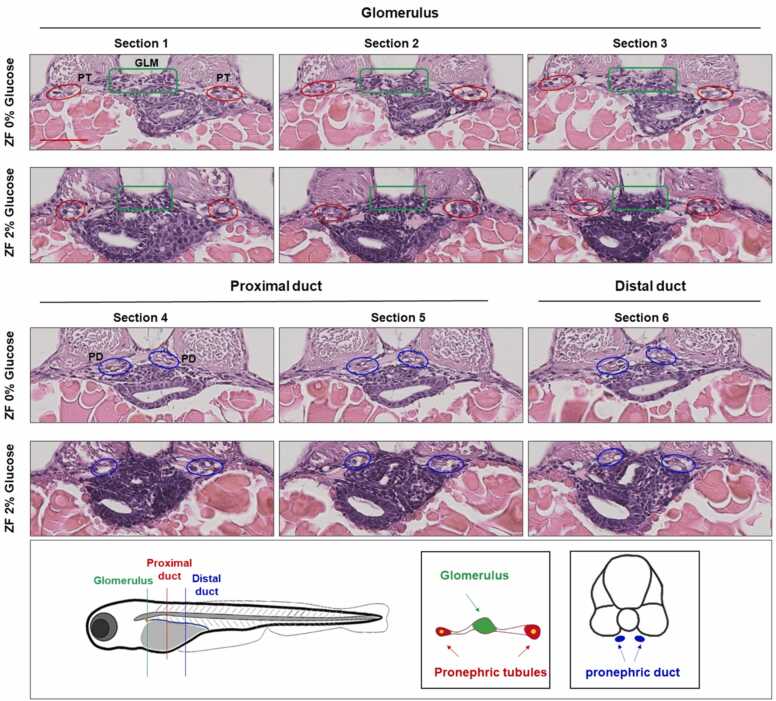
Hyperglycemia-induced pronephric abnormalities in zebrafish model. Representative micrographs from the level of the glomerulus (1, 2, 3), proximal pronephric duct (Sections 4–5), and distal pronephric duct (Section 6) are shown. Shapes indicate the location of the glomerulus (GLM, green), pronephric tubule (PT, red), and pronephric duct (PD, blue). Abbreviations: ZF, zebrafish.

The incubation of developing zebrafish embryos in 2% glucose resulted in increased total free-glucose levels in the whole-zebrafish homogenates. The average total free-glucose levels in the 3-day-old zebrafish group incubated in E3 media with 2% glucose measured 69.6 mg/dL compared to 43 mg/dL glucose levels in the control zebrafish group incubated in 0% glucose. Renal development in the zebrafish model was then evaluated. In accordance with the previously established hyperglycemia model [64], alterations to the developing renal system were noticed in the zebrafish incubated in 2% glucose conditions. Indeed, hyperglycemia caused noticeable tubular morphological changes to the glomeruli, pronephric tubules, and proximal and distal ducts, as evident in the examined histological sections, that were associated with the increase in total glucose levels (Fig. 8**).** The hyperglycemia-induced zebrafish model showed an abnormally distorted pronephros: the pronephric tubules were reduced in size at the proximal and distal pronephric ducts at 2% glucose condition when compared to the control group at 0% glucose.

**Fig. 9:**
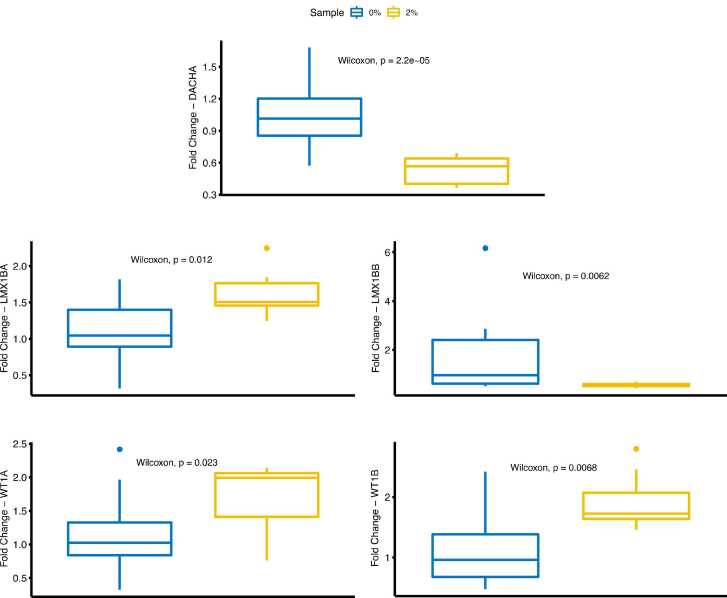
Expression of key TF genes in hyperglycemia-induced zebrafish model. Expression levels of key TF genes (A) *dach1a*, (B) *lmx1ba*, (C) *lmx1bb*, (D) *wt1a* and *(E) wt1b* were determined using quantitative real-time PCR and normalized to the internal control gene (*eefa1*), and relative expression was calibrated using the mean expression of control samples. Data are expressed as mean± SEM. Groups were 0% glucose and 2% glucose conditions.

We next examined the effects of hyperglycemic conditions on the expression of DACH1A (zebrafish ortholog: *dachd*), LMX1B (zebrafish orthologs: *lmx1ba* and *lmx1bb*), and WT1A (zebrafish orthologs: *wt1a* and *wt1b*). DACH1 and WT1 were prominent in the DEG and MI analyses, and LMX1B was important in the MI analysis and confirmed by STRING network (Fig. 3, Fig. 7). The evaluation of expression profiles of the key TF genes in the hyperglycemia-induced model demonstrated that, with increasing hyperglycemia, there was a significant reduction in *dach1 and lmx1b* expression levels and a significant increase in *lmx1a*, *wt1a,* and *wt1b* orthologs (Fig. 9).

## 4 Discussion

High-throughput transcriptomics and analysis methodologies have made it possible to explore the mechanisms of complex diseases in detail. Finding the gene regulatory pathways, which are important and potentially causal to the phenotypic changes or pathogenesis of a disease and the DEGs, is challenging but worthwhile objective. In this study, we found distinct clusters of genes that were dysregulated in a broadly renal compartment-specific pattern using nine individual datasets of human DN and healthy control glomeruli and tubulointerstitial tissues. Specifically, most downregulated core DEGs defined within this study were strongly downregulated in the glomerulus compartment. In contrast, the core DEGs showed more pronounced upregulation in the tubule datasets. These patterns are in line with previous reports of gene-expression downregulation in glomerulus samples [70], [71], [72] and upregulation in tubule samples in DKD [71].

Using network models, we identified a small but a closely connected cluster of genes enriched for podocyte differentiation, several of which were identified as genes encoding critical TFs involved in podocyte function. Podocyte damage is one of the hallmarks of DN and leads to proteinuria and kidney damage [73], [74]. We selected a group of three TFs for functional validation in the hyperglycemic zebrafish model based on the combined outcomes from DEG and MI network analyses and their importance for podocyte differentiation. Hyperglycemia resulted in altered zebrafish pronephric development and was associated with altered expression levels of *dachd, lmx1b*, and *wt1.* Moreover, the occurrence of abnormal pronephric development adds to the substantial evidence that the key TF genes *LMX1B*, *DACH1*, and *WT1* are associated with hyperglycemia-induced nephropathy. Firstly, *DACH1* was consistently downregulated across all our analyzed datasets, presenting as a core downregulated DEG in human kidney tissue and a downregulated gene (*dachd*) in the hyperglycemic zebrafish model, consistent with its downregulation in human nephrectomy samples [68] and its knockdown effect (using *dachd* ortholog) on compromised podocyte function in zebrafish [75]. DACH1 expression is localized to the glomerular podocytes and epithelium of convoluted tubules during kidney development [76] and the nuclei of glomerular and distal tubular epithelial cells in adult kidneys, but not to the renal interstitium of normal tissue [77]. DACH1 functions as a TF and a key cell-fate determinant [78] which is critical for proper kidney development and function [79]. For example, it was demonstrated to protect podocytes (by podocyte-specific augmentation, [68]) and tubules [68] during experimentally induced kidney injury. Moreover, global knockouts of *Dach1* in mice lead to early postnatal death [68], [80], and kidney hypoplasia suggesting global *Dach1*-KO mice die from renal failure [68]. Immunohistochemical staining of DACH1 proteins in human renal biopsies of immunoglobulin A nephropathy (IgAN) and idiopathic membranous nephropathy (IMN) patients show significantly decreased eGFR with declining DACH1, and significantly increased urinary protein in IMN with increasing DACH1 [77]. Although the biological mechanism behind DACH1 involvement in adult kidney tissue remains a mystery, the application of DACH1 as a predictor for clinical parameters [77] in CDKs such as IgAN and IMN highlights its potential as a key molecule in DN and lays the groundwork for future investigations into its mechanistic role in disease progression.

*LMX1B* was identified as a second core DEG that was downregulated in the meta-analysis datasets for glomeruli and tubules. Similarly, *WT1* (a core DEG) was consistently downregulated in glomeruli and in the majority of tubule datasets. The roles of WT1 and LMX1B in organ development and renal pathophysiology are well established. Multiple studies have shown that WT1 is involved in the development of the kidneys, neurons, and genitourinary tissue [81], and LMX1B is similarly involved in the development of kidneys [82], neurons [83], [84], and limbs [82], [85]. WT1 has been implicated as an essential regulator of podocyte fate [86], and it also functions in the activation of the downstream target LMX1B [69], [84]. Like WT1, LMX1B also plays a significant role in podocyte differentiation and function. It has been reported that the loss of *LMX1B* function results in hypoplastic kidneys that lack mature glomeruli or nephrons [87]. Indeed, in *Lmx1b*-knockout mouse models, kidney size diminishes, and glomeruli do not fully differentiate [69].

In addition to the poor kidney morphology outcomes related to the expression of TFs described above, we found the strong enrichment of pathways related to other nephropathic complications and multiple renal-system development pathways. Specifically, we found evidence for the downregulation of several key experimentally validated genes in DN, kidney development, and podocyte function in the glomeruli, such as *FOXC1* [88], *MAGI2* [89], *PTPRO* [90], *NPHS1* [91], and *NPHS2* [92]. More specifically, *TYRO3* was included within the critical hub genes in the network analysis and was generally downregulated in our meta-analysis. *TYRO3* is a protein expressed in podocytes and reported to play a critical role in maintaining podocyte function, especially in DN [93]. It has also been reported that *TYRO3* mRNA expression decreases with the progression of the disease, and it may be a potential drug target [94]. The disease and gene-function enrichment analyses provided evidence of the association between DN and signature complications of CKD and other poor systemic outcomes, such as anemia, proteinuria, and humoral immune responses. Although DN is considered a non-inflammation sub-type [95] of nephrotic syndrome [96], we found there was sporadic but highly elevated expression of immune activation in gene-function aggregate 2. This aggregate included genes of notable significance to diabetic nephropathy, specifically in the tubulointerstitial compartment for *CXCL6* [97], [98], and for the key DN target *C3,* which was recently identified in a bioinformatic analysis [99] and was shown to be involved in complement-system mediated glomerular and tubular injury using the ZS rat model [100]. Although the relevance of *CXCL6* has been validated in in vitro using a human podocyte cell line [98] and in kidney biopsies and resections [97], the role of *C3* in remains poorly characterized in kidney tissue from patients with diabetic nephropathy. Overall, the enrichment of pathways relating to immune function supports the pathophysiology re-defining role of inflammation in diabetic nephropathy [95].

This study has potential limitations, which are highlighted by the presence of unexpected results. Although we expected all orthologs to be downregulated in our qPCR assay in agreement with our meta-analysis, we found unexpected upregulation in the zebrafish hyperglycemic validation model for both *WT1* orthologs and one of the *LMX1B* orthologs (*lmx1ba*). The experimental conditions we used (2% glucose treatment for 3 days) may not have been stringent enough to induce the DN pathology experienced by patients included in the datasets. Additionally, we chose to examine gene expression of the key TFs using whole-larvae homogenates—as opposed to pronephros tissue— as it was logistically more feasible. Using whole larvae for RNA extraction could have skewed the results on our target genes, as they are involved in many developmental pathways that could last for up to 6 weeks after embryonic development in zebrafish [101], during which time the proteins encoded by these genes are expected to be highly abundant. To address some of the difficulties raised here, experimental designs utilizing organ-specific assays, together with immunohistochemistry or in situ hybridization labeling for the major DEGs, would be necessary. Future studies should therefore employ more focused experimental setups and targeted expression assays in zebrafish models in order to further validate the role of key genes in CKD.

Finally, by providing gene-expression-supported evidence for the over-representation of genes related to immune responses, renal system development, and diabetic/CKD development and validating key-TFs with podocyte-specific functions, our study has highlighted the systemic nature of diabetic nephropathy as a chronic disease [102]. By utilizing multiple independent datasets and leveraging the power of meta-analysis, we could highlight DN driving key TFs that are typically hidden amid a sizable number of DEGs in any given study. As a result, this work identifies and offers a confined set of biomarkers that may have predictive and therapeutic implications for diabetic nephropathy.

## Declarations

### Ethics approvals and consent to participate

• IACUC ethical approval: Institutional Animal Care and Use Committee (IACUC). Protocol Number: EVMC-2020–006
• IACUC ethical approval: 2020–1132, Sidra Medicine Zebrafish Facility approval date: 06/15/2020, Expiry date: 06/15/2023.

## Funding

This work is supported by Sidra Medicine Research Branch, Qatar, grant number SDR100002. Author AAA is the project lead principal investigator, budget holder, and the recipient of the research funding support.

## Consent for publication

All authors have approved the manuscript and give their consent for submission and publication.

## Data Sharing Statement

Data used are publicly available.

## CRediT authorship contribution statement

Ikhlak Ahmed, and Mubarak Ziab chose and examined the final datasets needed to construct the manuscript, writing, visualizing, and editing the final manuscript. Sahar Da'as, designing the zebrafish model and tests, collecting data, interpreting and writing the outcomes of the research, and visualizing the results. Waseem Hasan, zebrafish experiments and evaluation. Elbay Aliyev data analysis and dataset search. Sujitha P. Jeya molecular biology experiments linked to zebrafish validation, and initial dataset search. Sabah Nisar, the final manuscript version was editing and illustration. Ajaz A. Bhat experimental lab work in connection with the validation studies. Khalid Fakhro critical final revision and editing. Ammira S. Alshabeeb Akil, budget holder, study design, lab staff supervision, manuscript writing and revision, obtained the final approval of all authors, and produced the final manuscript draft.

## Competing interests

The authors have no relevant financial or non-financial interests to disclose.

## Data Availability

in addition to the publicly available datasets, Materials described in the manuscript, including all relevant raw data, will be freely available to any scientist wishing to use them for non-commercial purposes.
